# Exploring Risk, Antecedents and Human Costs of Living with a Retained Surgical Item: A Narrative Synthesis of Australian Case Law 1981–2018

**DOI:** 10.2147/JMDH.S316166

**Published:** 2021-08-31

**Authors:** Sonya R Osborne, Tina Cockburn, Juliet Davis

**Affiliations:** 1 School of Nursing and Midwifery, Faculty of Health, Engineering and Sciences, Centre for Health Research, Institute for Resilient Regions, University of Southern Queensland, Ipswich, Queensland, Australia; 2 Australian Centre for Health Law Research, Faculty of Business and Law, Queensland University of Technology, Brisbane, Queensland, Australia; 3 Griffith Criminology Institute, Griffith University, Mt Gravatt, Queensland, Australia

**Keywords:** unintended retained foreign object, retained surgical item, retained surgical instrument, retained surgical sponge, gossypiboma, sentinel event, adverse event

## Abstract

**Objective(s):**

This study aimed to critically examine the circumstances contributing to, and the human costs arising from, the retention of surgical items through the lens of Australian case law.

**Design, Setting and Participants:**

We reviewed Australian cases from 1981 to 2018 to establish a pattern of antecedents and identify long-term patient impacts (human costs) of retained surgical items. We used a modified four-step process to conduct a systematic review of legal doctrine, combined with a narrative synthesis approach to bring the information together for understanding. We searched LexisNexis, AustLII, Coroner Court websites, Australian Health Practitioner Regulation Agency Tribunal Decisions and Panel Hearings, Civil and Administrative Tribunal summaries, and other online sources for publicly available civil cases, medical disciplinary cases, coronial cases, and criminal cases across all Australian jurisdictions.

**Results:**

Ten cases met the inclusion criteria, including one coronial case, three civil appeal cases, and six civil first instance cases. Time from item retention to discovery ranged from 12 days to 20 years, with surgical sponges the most frequently retained item. Five case reports indicated possible deviations from standard protocols regarding counting procedures and record-keeping. In the four cases that reported on count status, the count was deemed correct at the end of surgery. Case reports also showed the human costs of retained surgical items, that is, the long-term impacts on patients associated with a retained surgical item. In eight of the nine civil cases, ongoing pain was the most frequently reported physical symptom; in three cases, patients suffered psychosocial symptoms requiring treatment.

**Conclusion:**

While there was little uniformity in the items retained or how items came to be retained, we identified significant time delays between item retention and item discovery, coupled with long-lasting physical and psychosocial harms suffered by patients living with a retained surgical item. Current prevention strategies, including national standards-based professional practices, are not always effective in preventing retained surgical items. An internationally standardised taxonomy and reporting criteria, more consistent reporting, and open access to event and risk data could inform a more accurate global estimate of risk and incidence of this hospital-acquired complication.

## Introduction

The total global volume of surgical operations performed in 2012 was estimated at almost 313 million procedures,^1^ and the rate is undoubtedly increasing as the burden of disease requiring interventional surgery increases.^2^ In the same year, the International Surgical Outcomes Study Group estimated an in-hospital surgical complication rate of 16.8%.^3^ From this, we can extrapolate that over 50 million patients will suffer from a surgical complication in their lifetime. Comparatively, the incidence of in-hospital surgical complication in Australia and New Zealand was reported to be 20% in 2013,^4^,^5^ which was higher than the international average. More recently, a New Zealand study found that 40% of patients reported experiencing a surgical complication,^6^ another indication that surgical complication rates may be rising.

Although surgical complications seem ubiquitous, adverse events, which result in harm to a person receiving care, are potentially preventable. One such adverse event is when a surgical item is unintentionally left behind in the patient after surgery, also known as a retained surgical item (RSI). In most jurisdictions around the world, an RSI is a reportable adverse event. We previously reported findings from this review in our analysis of the key legal issues arising from RSI claims for compensation and the phenomenon of the vanishing trial in Australia.^7^ In this paper, we focus our attention on understanding the risks, antecedents, and human costs of living with a retained surgical item and make recommendations to improve detection, responses and reporting.

## Background

### Risk and Prevention of Retained Surgical Items

Over the last decade, common risk factors for RSIs have been reported in the international literature,^8–14^ and the list is growing. For example, in 2018, Steelman et al examined 319 event reports of retained surgical sponges submitted to the Joint Commission in the United States of America (USA) and identified more than 1400 contributing factors across eight broad categories, with most relating to human factors (interaction between humans, such as staff orientation and supervision; medical staff credentialing and peer review; staffing levels and skill mix), leadership (eg policies and procedures and compliance; nursing and medical leadership; and organisational culture) and communication (eg oral, written and electronic; and with doctors, with administration, and among staff).^15^

Prevention strategies are consistent around the world and supported by national professional organisation standards for practice, or local policies and procedures. Strategies range from manual counting of accountable items to reconcile baseline counts (undertaken before incision) with final counts (undertaken before wound closure); methodical wound exploration prior to wound closure; clear processes to be undertaken in the event of an incorrect surgical count, such as searching in the patient, in and around the aseptic field, and in the operating room environment for the missing item; use of radiographs of the operative site to locate the missing item; and effective communication among the surgical team.^16^ Surgical teams routinely rely on discrepancies (for example, an incorrect count) in the manual surgical count procedure as a prevention strategy to identify situations of potential or actual RSIs. However, evidence suggests that sole reliance on manual counting procedures and radiographs (x-rays) are inadequate prevention strategies. Large seminal trials estimate that manual counting procedures are only 77% effective in picking up an RSI^17^ and intraoperative x-rays are only 67% effective in picking up RSIs.^18^ Furthermore, in 62–88% of RSI cases, the count at the end of the procedure was actually reported as correct.^10^,^18^,^19^ In the past decade, several adjunctive technologies have been incorporated into prevention strategies, such as radio frequency identification (RFID), bar coding of surgical items or other automated counting technologies;^20–22^ however, none of these newer technologies are used consistently across jurisdictions or facilities.

### Global Incidence and Prevalence of Retained Surgical Items

Quantifying the incidence and prevalence of RSIs is problematic. The most frequently quoted estimates to date of the incidence of RSIs from the published literature range from 1 in 5500 to 1 in 18,760 in-patient operations.^10^,^17^,^18^ Around the world, the true incidence is difficult to accurately quantify due to inconsistencies in reporting criteria and reporting requirements. The Organisation for Economic Co-operation and Development (OECD), an intergovernmental economic organisation of 37 member countries, reports annually on key indicators for population health and health system performance. In 2017, the OECD reported an average rate in 2015 for a foreign body left in during a procedure was 5.4 per 100,000 surgical discharges, ranging from 0.2 per 100,000 (Poland) to 12.3 per 100,000 (Switzerland).^23^ In the 2019 data, the average rate had decreased slightly to 5.2 per 100,000 separations.^24^

Attempts to quantify incidence or prevalence of RSIs have historically been drawn mainly from studies of incident reports and, in some cases, medical insurance claims. It has long been established that adverse events are underreported and studies in the last decade continue to support this finding. A retrospective study^25^ of 5375 patient records in 14 hospitals in the Netherlands compared adverse events found in the patient records against the four main mechanisms of reporting: informal patient complaints, formal patient complaints, incident reports submitted by health professionals, and medico-legal claims filed by patients. Of the 498 adverse events identified in the patient records, only 18 (3.6%) were found in one or more of the four reporting systems.^25^

### Retained Surgical Items and the Australian Context

In 2004, Australian Health Ministers agreed on a national core set of eight sentinel events requiring mandatory reporting by all Australian public hospitals,^26^ with RSIs being one of the eight. Comparatively, the incidence of RSIs in Australia is higher than the international OECD average, with a reported rate in Australia in 2015 of 8.8 per 100,000 surgical admissions,^23^ decreasing to 8.2 per 100,000 surgical admissions in 2017.^24^ In the ten years between 2005/2006 and 2015/2016, 322 incidents of RSIs requiring re-operation or a further surgical procedure were reported by Australian hospitals.^27^ In Australia, the true incidence and prevalence is also difficult to accurately quantify due not only to inconsistencies in national reporting requirements but also inconsistencies in the types of organisations that are required to report. For instance, mandatory reporting does not apply to private facilities in all states (see Supplementary Materials Table S1). Individual state and territory government reports detail events and circumstances, usually explored by root-cause analysis, as possible contributors to retention in specific cases. While these reports provide a useful snapshot of actual reported incidents, they contain limited detail on antecedents for retention or on the longer-term impacts on patients.

Discovery of an RSI usually occurs while the patient is still in hospital or shortly after discharge. Despite international, state and territory government reports compiled from mandatory reporting, we still know little about the antecedents to items being retained or the unintended and long-term consequences of RSIs. Other publicly available data sources, such as case law reports, could provide more and different information that may assist in accurately quantifying the true incidence and risk and allow us to fully appreciate the aftermath and long-term consequences of RSIs.

With this in mind, a review of legal cases brought before a court or tribunal has the potential to offer valuable additional insights that may contribute to the collection of prevention measures currently in place. These cases may provide supplementary insight into the factual circumstances, antecedents, and impacts of retention, given that detailed information is required for determining legal responsibility and personal and economic damages. Thus, the purpose of this study was to describe a methodology for reviewing legal documents and critically examine the circumstances contributing to, and the human costs (long-term patient impact) arising from, the retention of surgical items through the lens of Australian case law.

## Methods

We adopted the four-step process for conducting a systematic review of legal doctrine described by Baude et al^28^ to enable better analysis of claims made about legal doctrine and reduce actual or perceived researcher bias. The four steps for conducting the systematic review were: (a) establishing a clear and precise legal question, (b) defining a sample of cases, (c) explaining how cases will be weighted, and (d) critically analysing the cases to inform a stated conclusion.^28^ A protocol for this review has not been previously published.

### Legal Questions Guiding the Critical Case Review

The research questions guiding the review were:

(1) What are the material factual circumstances of cases concerning RSIs in Australian hospitals brought before Australian courts and tribunals from 1981 to 2018?

(2) Can a pattern of antecedents for risk of RSIs be established from analysing case law to:

- determine a more accurate estimate of patient risk, and
- offer insight into additional strategies for reducing risk or prevention?

(3) What are the long-term impacts on patients associated with an RSI?

### Sample of Cases and Search Strategy

Cases were included in the sample if they met the following inclusion criteria: civil claims, criminal cases, medical disciplinary cases, and coronial court cases from 1981 to 2018 from Australian jurisdictions concerning incidents of RSIs in Australian hospitals. The search start date was 1981 because national guidance for nurses working in the operating room for the management of accountable items used during surgery was first published in 1980 by the professional body then known as the Australian Confederation of Operating Room Nurses.^29^ Cases were excluded if a surgical item was intentionally retained and later removed without incident and no harm was attributed to that item.

Using variations of the search terms surg* OR medical AND retain* OR “adverse event” AND count and related words, the following publicly available data sources were searched for the period 1981–2018: LexisNexis (searches for Australian case law); Australasian Legal Information Institute (AustLII) (searches of state and territory Professional Regulatory Boards); Coroners’ Courts for each State and Territory (for summaries of Coronial Cases); Civil and Administrative Tribunal Decisions in all jurisdictions (for health practitioner case summaries); and the Australian Health Practitioner Regulation Agency (AHPRA) Medical Board and Nursing and Midwifery Board Panel Tribunal Hearings (for health practitioner case summaries).

We sought to consider all online cases relating to the research questions within the relevant period; however, the disparate nature of these online sources meant that the chronological cut-off for the online availability of legal cases varied across platforms. The full search strategy parameters, brief descriptions of the key databases searched, and an example of the search string used in LexisNexis can be found in the Supplementary Materials Tables S2–S4.

### Weighting of Included Cases

As we had no preconceived expectations of how many or what type of cases would be found, cases were equally weighted. However, following the legal doctrine of precedent, which provides that similar cases should be decided in similar ways and achieve similar outcomes, it could be appropriate to give cases whose reasoning is partly rejected or disputed by the courts in subsequent cases less weight in the final analysis, and give those cases which were considered and followed in subsequent cases more weight.

### Method for Critical Case Analysis

Following a systematic search of case law, the included cases were reviewed by a university law professor (TC) with experience in civil medical litigation and case law review and cross-checked by the Project Law Research Assistant (JD). Key case characteristics were extracted, and a coding framework was settled upon by the research team (TC, JD, SRO). The cases were then coded, critically analysed and synthesised to draw out key trends. These trends were then expanded into narrative summaries of the relevant facts and law in each case and discussed by the research team. Details of the data extracted can be found in the Supplementary Materials Table S5.

This approach to legal doctrine review was strengthened by using a narrative synthesis approach, which relies mainly on the use of words and text to summarise and explain the findings from the included cases. Although originally described for use with systematic reviews of intervention effectiveness or factors influencing the implementation of interventions, we adopted the general framework for narrative synthesis described by Popay et al^30^ to “tell the story” of the findings from the included cases. The four main elements of the narrative synthesis framework were: (1) developing a theory of how, why and for whom the prevention interventions work (or in the case of RSIs, did not work), (2) developing a preliminary synthesis of findings, (3) exploring relationships in the data, and (4) assessing the robustness of the synthesis for drawing and generalising conclusions.

The theory underpinning our narrative synthesis is James Reason’s accident causation model,^31^ which proposes that in complex systems multiple barriers or layers exist to prevent accidents and errors and that failure in the system can occur if the plan is adequate but associated actions are not deployed as intended or that the actions go as intended but the plan is flawed.^32^

## Results

**Figure 1:**
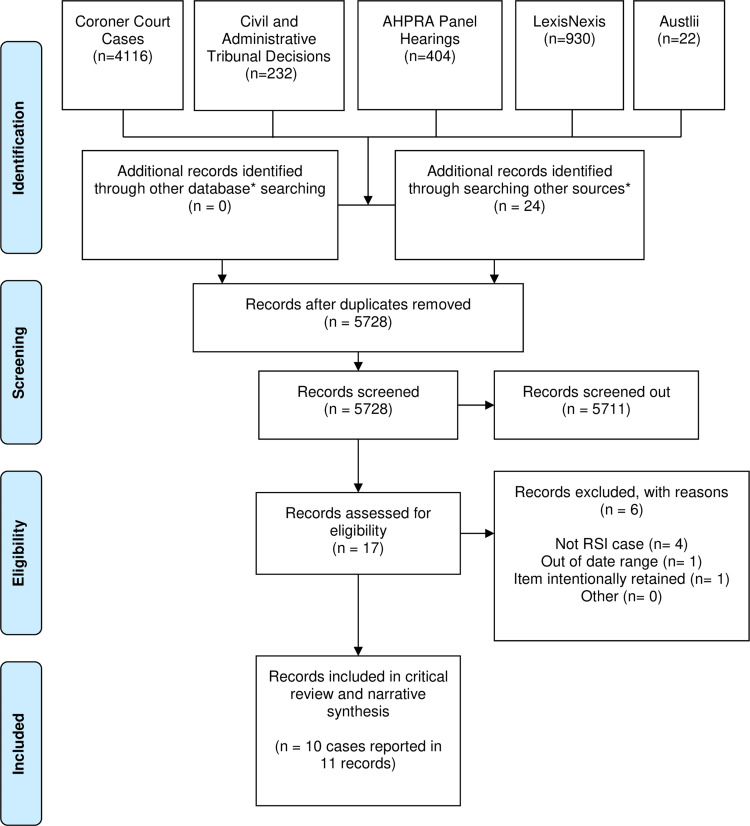
Australian case law flow diagram (diagram adapted from Moher D, Liberati A, Tetzlaff J, Altman DG. The PRISMA Group. Preferred reporting items for systematic reviews and meta-analyses: the PRISMA statement. *PLoS Med*. 2009;6(7):e1000097).^44^

As depicted in Figure 1, from a search pool of 5728 case records (after two duplicates were removed), only 11 decisions reporting on 10 cases^33–43^ were found concerning incidents of RSIs and meeting the inclusion criteria, including one coronial case,^43^ three civil appeal cases,^33^,^34^,^39^ and six civil first instance cases,^35^,^38^,^40–42^ including two decisions referring to the same legal matter.^36^,^37^ Despite the small sample of cases available, it is possible to derive a number of observations about how RSI claims are considered in the Australian legal system. It should be noted that the majority of the 10 cases located are unreported, with only two involving a final consideration of liability and damages.^33^,^39^

Most cases reviewed were procedural, which means that the plaintiff (usually, this was the patient or patient’s family or estate) sought the Court’s permission (called “leave” in legal terms) to bring an action, usually against the surgeon, the nurses, and/or the hospital/health service organisation, outside the limitation period (including an appeal against the dismissal of a matter),^34^ or to amend their previous statement of claim based on new evidence.^35^,^38^ Under Australian law, a statute of limitation restricts the time within which a person (the plaintiff) can commence proceedings and a medical negligence case cannot generally be brought after three years from the date on which the cause of the action was discoverable to the plaintiff.^45^

A brief summary of the findings of key characteristics from each of the 10 included cases are presented in Table 1. A more detailed summary of findings table, including the material factual circumstances of the cases, antecedents for risk, and long-term impacts, can be found in the Supplementary Materials Tables S6 and S7.

**Table 1:** Summary of Key Findings Table (Abbreviated)

Case Citation [Date, State]	Type of Hospital	Date of Retention (ie, Date of Surgery)	Type of Surgery	Item (s) Retained	Means of Discovery	Date of Discovery *[Disclosure]*	Time from Retention to Discovery/Removal
*Elliott vBickerstaff* [1999, ACT]^33^	Private	13 Jun 1991	Total hysterectomy and colpo-suspension	Sponge	Patient complained of “physical problems”	“about six weeks later”	“about six weeks”
*Gaynor vMilton; Ulladulla Hospital* [1981, NSW]^34^	Public	10 Jun 1975	Appendicectomy	Piece of forceps	Item known to be retained; confirmed with x-ray	*[Authors Note: Date of discovery unclear]*	*[Authors Note: Details missing from record]*
*Hughes vMinister for Health East Pilbara Health Service* [1999, WA]^35^	Public	20 Dec 1994	Insertion drainage tubes	Drainage tube	Patient complained of physical symptoms (severe central abdominal pain, nausea, vomiting, constipation, fatigue); item found by x-ray and ultrasound scan	21/22 Dec 1994 - item missing; 19 January 1995 - retention of item in abdominal wall discovered	28 days
*Ives vAustralian Capital Territory and Anor* [1995, ACT]^36^/*The Australian Capital Territory v* *Ives* [1996, ACT]*37*	Public	“On or around 12 Mar 1974”	Securing/removing drainage tube in connection with hysterectomy	Straight needle	Patient had chest and spinal x-rays for unrelated condition; item revealed	11 Oct 1994	20 years, 7 months *[Authors Note: item not removed due to greater perceived risk]*
*Kenjar vAustralian Capital Territory* [2014, ACT]^38^	Public	26 Aug 2008	Open reduction, multiple K-wire fixation of right hand	Piece of K-wire	Patient had pain, swelling, necrotic tissue, abscess in right hand; x-ray taken days after debridement surgery revealed item	2 Oct 2008 *[Authors Note: Patient not informed of retention after initial surgery]*	16 days
*Langley & Warren vGlandore Pty Ltd & Thomson* [1997, QLD]^39^	Private	22 Feb 1990	Total abdominal hysterectomy	Sponge	Patient had “painful symptoms” following surgery; subsequent surgery revealed item	“some ten months later”	“some 10 months later”
*Miller vBroadbent* [1999, QLD]^40^	Private	Oct 1992	Laparoscopy stomach banding	Silicon tubing	Patient had ongoing abdominal pain; item revealed during exploratory surgery to identify cause of pain	5 June 1996	3 years, 8 months
*O’Hagan vSakker* [2011, NSW]^41^	Private	10 Aug 1992	Hemi-colectomy/ sigmoid colectomy	Sponge	Patient admitted following fall; complained of abdominal pain; x-ray taken; item revealed	2 Oct 2007 *[Authors Note: Patient only became aware of RSI after removal]*	15 years, 1 month
*Smith vMarcus* [1989, NSW]^42^	Public	24 Nov 1977	Hysterectomy and insertion of drainage tube	Drainage tube	Patient had persistent pain and discomfort in the stomach and pelvic area exacerbated by walking. Eventually, had IVP examination, item present on film but not in report; IVP film later re-examined by GP, item confirmed by ultrasound and CT scan	24 Nov 1987 *[Authors Note: Patient not aware of RSI previously]*	10 years
*Investigation into Death of* *James Stirling McKinlay* [2013, TAS]^43^	Public	2 Jun 2012	Follow up surgery for internal bleeding post pancreaticoduodenectomy	Sponge	Multiple surgeries: item intentionally retained to be removed at subsequent surgery; item not found; x-ray and later CT scan taken; item visible on both films but not in either report; item revealed during subsequent surgery	14 June 2012	12 days

### Material Factual Circumstances of Cases Concerning Retained Surgical Items

#### Types of Surgery and Items Retained

The legal cases revealed little uniformity in the items retained as presented in Table 1: silicon tubing in the abdominal cavity retained during a laparoscopy stomach banding operation,^40^ Kirschner-wire (K-wire) fragment retained in the right hand after an open reduction and multiple K-wire fixation,^38^ one instance of a drainage tube retained after a recurrent umbilical hernia,^35^ and another after a hysterectomy,^42^ a straight needle, which had migrated into the heart after being retained during a hysterectomy^36^,^37^ a broken piece of forceps retained in the body after an appendicectomy,^34^ one instance of a surgical sponge being retained in the patient’s abdominal cavity at the conclusion of a colectomy,^41^ two instances of a sponge being retained after the patient underwent a hysterectomy,^33^,^39^ and a final instance of a sponge being accidentally retained after being initially left in situ deliberately to stem intra-abdominal bleeding.^43^ While the majority of cases involved open abdominal or pelvic surgical procedures (n = 8), one case was a minimally invasive abdominal surgical procedure, and one case was an orthopaedic upper limb procedure. The most frequently retained item was the surgical sponge, which occurred in 4 of the 10 cases.

#### Means of Discovery and Disclosure of Retained Surgical Items

Time from retention to discovery of RSIs ranged from 12 days to 20 years with significant disparity in the manner of discovery of the retained item across the cases (see Table 1). In most cases, the discovery came after the patient presented with physical symptoms. In one case,^36^,^37^ a retained straight surgical needle was discovered incidentally after a chest x-ray for an unrelated condition; and in another, a retained surgical sponge was discovered after presentation to the emergency room following a fall.^41^ In two other cases, the RSIs were device fragments that were known to be retained at the time of the surgery; a broken forceps tip in *Gaynor v Milton*^34^ and a broken piece of a K-wire in *Kenjar v* *Australian Capital Territory* .^38^

A notable feature in three of the reviewed cases was a failure to identify a retained item that was visible on postoperative x-ray scans taken at the time of the suspected missing item. In *Kenjar v* *Australian Capital Territory*,^38^ the patient underwent day surgery for an open reduction and multiple K-wire fixation to his right hand on 26 August 2008, and a later surgery on 16 September 2008 to remove the K-wires. Images taken during the earlier surgery revealed a fragment of K-wire retained in his right hand, but no action was taken to remedy this until the patient returned to hospital with pain and swelling in his right hand, necrotic skin, and an abscess on 30 September 2008, 14 days later. In *O’Hagan v* *Sakker*,^41^ the patient, who suffered from longstanding abdominal and pelvic problems, underwent a partial removal of her colon on 10 August 1992 and consequently experienced fevers, abdominal cramps and loss of bowel control. She had an abdominal x-ray on 7 June 2003 in anticipation of a planned colonoscopy procedure. This x-ray film showed the retained surgical pack in the patient’s abdominal cavity; however, the Court accepted that she was not informed of this x-ray finding in 2003, when it was initially examined. The patient underwent an abdominoplasty in February 2005 and a further colonoscopy in February 2007; however, there was no evidence that x-rays were taken or viewed for these surgeries. The foreign body, which by the time of its removal was “about the size of a grapefruit”, was only discovered in late September 2007 when the patient was admitted to hospital suffering from abdominal pain after falling several days earlier. In the Tasmanian Coroners Court matter of the *Investigation into Death of James Stirling McKinlay* (2013),^43^ the retained pack was visible on an x-ray taken on 6 June 2012, but the radiologist did not report it, and managing doctors did not see it. The retained pack was visible in a CT scan of the abdomen on 7 June 2012, but again it was not noted. The retained pack, which was tightly compressed and separately located from the other packs, was discovered and removed during another operation on 14 June 2012.

In *Hughes v* *Minister of Health*,^35^ the discovery of a retained object was hindered by postoperative care failures. The patient underwent surgery in September 1993 to repair a recurrent umbilical hernia. In a later surgery, two drainage tubes were inserted to drain fluid build-up. These drainage tubes protruded from the patient’s abdomen and were connected to a fluid suction apparatus. On 20 December 1994, the drainage suction apparatus was removed, as were stitches that held the drainage tubes in place. The drainage tubes remained in place, extending approximately 20 mm from the patient’s abdomen. On 22 December 1994, the left-side drainage tube was found to be missing. Despite this discovery, the plaintiff was discharged from the hospital after the removal of the right-side drainage tube. After discharge, the patient suffered from “severe central abdominal pain, nausea, vomiting, constipation and fatigue and was incapable of working”.^35^ X-rays and an ultrasound scan taken in early 1995 located the lost drainage tube within the anterior abdominal wall.

### Antecedents for Risk of Retained Surgical Items

While information about antecedents for item retention is limited in some of the reviewed cases, a number of cases in the sample reflect current literature on contributing influences related to human factors, such as deviations from protocols and poor or no communication between health professionals.

#### Human Factors – Deviation from Standard Protocol

The review considered whether operating room staff involved in the litigated procedures had performed appropriate procedural steps and checks in relation to the management and accountability of surgical supplies and equipment. Deviation from established protocols regarding counts and record-keeping was implicated in five cases. Only five case reports discussed counts and contemporaneous record-keeping in any detail. In four cases reporting on count status, the count was deemed correct at the end of surgery (see Table 2).

**Table 2:** Count Status at Key Timepoints in the Counting Procedure

Case Citation/Date	Item (s) Retained	Initial Count	Wound Closure Count	Skin Closure Count	Xray Taken
*Elliott vBickerstaff* [1999, ACT]	Sponge	Not recorded in case note	Not recorded in case note	Correct	Unable to determine if x-ray was taken
*Gaynor vMilton;* *Ulladulla Hospital* [1981, NSW]	Piece of forceps	Not recorded in case note	Item known to be missing	Item known to be missing	Yes, later (+)
*Hughes vMinister for Health East Pilbara Health Service* [1999, WA]	Drainage tube	Not recorded in case note	Not recorded in case note	Not recorded in case note *[Authors'Note: tube known to be missing* *day after stitches removed]*	Yes, later (+)
*Ives vAustralian Capital Territory and Anor* [1995, ACT]	Straight needle	Not recorded in case note	Not recorded in case note	Correct	Yes, much later and unrelated (+)
Kenjar v *Australian Capital Territory* [2014, ACT]	Piece of k-wire	Not recorded in case note	Not recorded in case note	Not recorded in case note	Yes, later (DOS) (+)
*Langley & Warren vGlandore Pty Ltd & Thomson* [1997, QLD]	Sponge	Not recorded in case note	Not recorded in case note	Correct	Unable to determine if x-ray was taken
*Miller vBroadbent* [1999, QLD]	Silicon tubing	Not recorded in case note	Not recorded in case note	Not recorded in case note	Yes, later (-); later exploratory surgery (+)
*O’Hagan vSakker* [2011, NSW]	Sponge	Not recorded in case note	Not recorded in case note	Correct	Yes, 2003 xray (+) but reported (-); 2003 xray re-examined later (+)
*Investigation into Death of* *James Stirling McKinlay* [2013, TAS]	Sponge	Not recorded in case note	Item intentionally retained	Incorrect - intentional retention	Yes, later, misread (-); later CT scan misread (-); later exploratory surgery (+)
*Smith vMarcus* [1989, NSW]	Drainage tube	Not recorded in case note	Not recorded in case note	Not recorded in case note	Yes, later, several xray reported (-); xrays and IVP re-examined later (+)

**Notes:** (+) Retained item found on x-ray; (–) Retained item not found on x-ray.

**Abbreviations:** DOS, day of surgery; IVP, intravenous pyelogram.

In *Langley & Warren vGlandore Pty Ltd & Thomson*,^39^ a sponge was left inside the patient’s abdomen after a total abdominal hysterectomy. The surgeons were given general assistance by an instrument nurse and a circulating nurse employed by the hospital. The nurses were found to have made an error in tallying the number of sponges used, incorrectly balancing the number of sponges retrieved at the end of the surgery with the number opened during the procedure. In *Elliott v* *Bickerstaff* 33 it was inferred at trial that the nurses present at the surgery miscounted the number of sponges used and provided the surgeon with “unfounded assurances” that all items were accounted for, leading to the retention of a sponge in the patient’s abdominal cavity. In *Ives v* *Australian Capital Territory*,^36^ and its 1996 appeal on a procedural point,^37^ the court examined the retention of a straight needle in the patient’s ventricle, which was alleged to have migrated from her abdomen after a hysterectomy in 1974. Evidence was led about the “standard practice” of counting all needles at the end of the surgery and recording of the count reconciliation on a whiteboard by the nurse.

… There was no record of a needle having gone missing or having broken. If there had been, it would have been regarded as a serious event.^36^

This recital of usual practice was confirmed by a nurse who routinely assisted the defendant surgeon. There was, however, no record kept of reconciling the needle check as it was not usual practice to keep a permanent record of the count in 1974. In *O’Hagan v* *Sakker*,^41^ which concerned the retention of a surgical pack after a sigmoid colectomy, the defendant surgeon also led evidence about usual hospital practice and procedures as at the operation date in 1992. However, in the absence of documentation in the medical records, the evidence of the surgeon’s usual practice was treated with caution by the Court because

… most drivers of motor vehicles would assert that they invariably stop at red traffic control lights, yet common knowledge indicates that the work of red light traffic cameras tells a very different story.^41^

The fifth case concerning a retained surgical sponge, the Tasmanian Coroners Court inquiry into the death of *James Stirling McKinlay* (2013)^43^ specifically discusses the importance of easily accessible and consistent documentation. The court found that the deceased underwent a lengthy and complicated “Whipples procedure” on 15 May 2012 to remove a cancer of the bile duct. Between the date of surgery and 1 June 2012, he underwent multiple surgeries, which unsuccessfully sought to address internal bleeding. The operating room nurse’s report for a further surgery on 2 June 2012 recorded that one large pack and six small packs were deliberately left in position to stem intra-abdominal bleeding. After surgery, the patient was transferred, with his medical records and notes, to the Royal Hobart Hospital. Surgery was undertaken on 4 June 2012 and six packs were removed, but one pack was accidentally retained. While Coroner Pearce found that the retained pack did not contribute to the patient’s death, he found that the deceased was transferred to the Royal Hobart Hospital with an incomplete medical record, which failed to formally communicate the number of packs left in situ on the handover. The Coroner recommended that because the count procedure is used as a risk mitigation strategy, it requires due diligence and care to ensure that the recording of the count is accurate, consistent between nursing and medical team members, and easily accessible as a communication tool, not only between clinicians but also between facilities when patients are transferred.^43^ The Coroner also made the following recommendation:

Each hospital should also consider whether a practice of abdominal x-ray following emergency abdominal surgery to identify and reduce the risk of retained packs might be appropriate.^43^

In all of these cases, the procedures described correspond with current ACORN Standards for Perioperative Nursing in Australia, which state that

[a]ll members of the operating or procedural team have a duty to collaborate to ensure that all items used during surgery and procedures are retrieved … accounted for and appropriately documented. (p75)^16^

#### Human Factors – Communication, Verbal and Written

Judgments in many cases linked deviations from the protocol closely to either inadequate verbal communication or written communication in the patient records. In two of the four cases concerning a retained surgical sponge, the count was communicated and documented (according to medical records) to be correct at the end of the surgery.^33^,^39^ In one case, the correct count was implied from the trial transcripts, despite a lack of written records confirming this.^41^ In either case, the presence or absence of written records impacted on the success of the plaintiffs’ or defendant’s case. For example, in *O’Hagan v* *Sakker*,^41^ the judge commented on the expectation of certain documents contained in the medical record to be able to provide evidence

… whether or not the relevant items were counted at the conclusion of the operation, and whether such counting was the subject of the signing off, in conformity with the usual practice.^41^

The cases in this sample underline the importance of clear and accessible communication, both verbal and written, as a safeguard to preventing RSIs.

### Harm Suffered and Unintended Consequences

Eight out of ten records reported harm suffered by the patient as a consequence of a retained surgical item. Physical harm was described in two cases.^39^,^40^ In five cases, a range of both physical harms and psychosocial harms were described;^33^,^35^,^38^,^40^,^41^ although in one of these the physical symptoms were masked due to multiple existing co-morbidities and were re-investigated after the patient presented to the emergency department for an unrelated fall.^41^ In one case, there was no mention of physical harm prior to discovery; however, psychosocial symptoms manifested after the retained item was discovered on a chest x-ray taken for an unrelated reason.^36^,^37^

It is important to note the potential for psychosocial harm as a corollary of a lengthy retention as evidenced in the following cases. In *O’Hagan v* *Sakker*,^41^ the patient suffered from ill-health and pain most of her life and had undergone multiple operations in an attempt to improve her quality of life. Evidence was tendered that as a consequence of the discovery and removal of a retained pack in her abdomen 15 years after the relevant surgery

… the plaintiff has become preoccupied with, and focussed upon, what she considers to have been the deleterious effects upon her health as a result of the pack having been left in her abdominal cavity. She has been preoccupied with psychological problems.^41^

Similarly, the patient in *Ives v Australian Capital Territory* became “depressed and anxious” after learning about the presence of an “extremely long” and fractured needle in her heart ventricle, which had migrated from her abdomen after being retained there more than twenty years earlier.^36^ In *Elliott v* *Bickerstaff*,^33^ the patient developed “ongoing psychological and physical problems” as a result of the six-week retention of a sponge in her abdomen. In the case of *Smith v* *Marcus*,^42^ the plaintiff endured constant pain, soreness and discomfort in the pelvic and stomach region, exacerbated by walking. After 10 years of persistent pain, multiple visits to a range of medical practitioners ordering a myriad of diagnostic tests, the cause was later discovered to be a retained drainage tube, determined to be in situ 10 years after surgery. Apart from the apparent physical harm in this case, psychosocial harm manifested in the patient’s feeling of self-doubt after years of being told that there was nothing wrong with her. The Court assessed that the patient was:

… a relatively unsophisticated lady who understandably seems to have adopted the attitude that whatever the cause of her problems a variety of skilled doctors after testing could detect nothing wrong and that she should learn to live with her ongoing discomfort.^42^

The plaintiffs (patients) in all cases suffered from harm post-surgery, regardless of the type of surgery, the item retained, or the length of time from retention to discovery; with psychosocial harm manifesting more in cases where the patient complained of ongoing physical pain but whose complaints were dismissed or in those patients living with an RSI once they became aware of the presence of the item and potential worse outcomes they could have suffered.

## Discussion

### Supplementing Existing Retained Surgical Item Data Sets by Analysis of Australian Case Law

It is well accepted in the academic and popular literature that reported incidents of RSIs are considered the “tip of the iceberg” when looking at the true extent of the problem in hospitals around the world. This may be due to the current absence of mandatory reporting of “near misses” and failures or delays in discovering RSIs due to patients who may be asymptomatic or suffering from non-specific symptoms;^46^ that is, symptoms not initially linked to a prior surgical procedure. Furthermore, the number of incident reports for a specific event may not be a reliable reflection of the frequency of that event nor of the true risk of the event occurring. For example, following their study of a falls prevention program, Abujudeh et al warned that the prevalence of incident reports may be more a reflection of a particular organisational focus on reporting of particular incidents at that point in time.^47^ More concerning is the Grattan Institute Report on Strengthening Safety Statistics,^48^ which concluded that incident reports cannot be relied upon to benchmark performance over time or across organisations, or to help understand what types of adverse events or harm to patients are most prevalent. This may be because incident reporting is mostly voluntary; and, where mandatory, reporting criteria and definitions (such as “end of surgery”) are not always clear or consistent, resulting in inconsistency in measurement indicators. This therefore contributes to the possible underestimation of the actual risk of a patient leaving the operating room with an RSI.

The National Hospital Morbidity Database, published by the Australian Institute of Health and Welfare,^27^ provides a useful overview of the incidence of RSI retention, while a number of state government reports detailed circumstances that contributed to the retention of surgical items in specific cases. The range of factors at different levels of the process leading up to an RSI, from unsafe individual actions to latent hazard conditions within the organisational system, demonstrate the application of Reason’s accident causation model.^31^,^32^ Some of these incidents arose from procedural failures (eg operating staff’s non-adherence to the use of the instrument count sheet; reliance on memory to remove a surgical gauze at the end of a procedure; performance of an organ closure despite incorrect swab count; commencement of wound closure prior to the completion of the first surgical count), and some from communication failures (eg a failure to report a missing swab after the initial swab tally was found to be incorrect; failure to confirm removal of a pack inserted by the anaesthetist). Retention also arose from issues with surgical instruments or equipment (eg use of equipment with easily removable parts, equipment failure) and use of other ancillary equipment (eg incorrect reading of intraoperative or postoperative x-rays or other scans).

Government reports provide a useful glimpse of RSI incidents; however, findings from government reports of mandatory reporting are typically based on root cause analysis, which is inherently subject to human biases of the investigators, such as hindsight bias or attribution error, as they attempt to determine causal factors of an adverse event.^49^ The aim of our study was not to find the one cause, per se, of the RSI or to attribute blame. We took the stance recommended by Henriksen et al^49^ “to be fair and yield new knowledge” (p71). As such, our efforts were directed at the antecedent circumstances that existed for the operating room personnel before the item was retained to make sense of the previously unknown factors contributing to the retention. This study sought to examine the antecedent circumstances leading up to, and the human costs arising from, the retention of surgical items through the lens of Australian case law reports of legal proceedings relating to RSIs.

### Review and Synthesis of Australian Case Law

Our study involved a review of civil cases, medical disciplinary cases, coronial cases, and criminal cases across all Australian jurisdictions. Only ten original cases concerning incidents of retained surgical items were located, a very small number when compared with the 322 incidents of retained items requiring re-operation or a further surgical procedure reported by Australian hospitals in the years between the years 2005–2006 and 2015–2016.^27^

Despite the small sample of cases available, it was possible to derive a number of observations regarding the Australian legal system’s consideration of claims relating to RSIs, particularly in relation to: most commonly retained items; the length of delay between retention and discovery; antecedents to retention; the human costs of retention and risk prevention strategies. We found that surgical sponges made up the highest proportion of surgical items retained (40%). This not only aligns with previous studies but also continues to be confirmed in more recent studies of root cause analysis investigation reports.^50^

In their study of reports from 2010 to 2015, Hibbert et al found that nearly a quarter of the retained surgical items were discovered either immediately in the postoperative period or on the day of the procedure, while about 1/6 were only detected after 6 months, with the longest period being 18 months.^50^ As our study examined legal cases across a much longer time frame, we were able to uncover that the time between retention and discovery could be as long as 20 years.

From these cases, it is evident that retention of surgical items (which encompasses a diverse range of items) is a widespread phenomenon that cannot be attributed to a particular surgical practice or type of surgery. As discussed above, retention may be impacted by a number of human factors including failure to adhere to established risk mitigation processes, deficient communication and record-keeping,^50^ and issues surrounding postoperative care practices including omissions in clinical handover information or mis-reading or misinterpretations of postoperative diagnostic x-rays, where in some cases, retained items later determined to be visible on postoperative scans were not identified at the time of the scan. The human factors implicated in the reviewed cases were referred to by the judges in their decisions and recommendations to address failures in the system that enabled human factors failures. The cases also revealed physical and psychosocial harms allegedly experienced by patients due to retention of the surgical item. Some of these harms were exacerbated by a lengthy delay before discovery, and most were certainly not known or expected at the time of transfer from the operating room or even prior to discharge from hospital.

Clark and Oakley^51^,^52^ argued that patients should be provided with comparative information about surgeons’ performance as part of the informed consent process (which is a universal pre-requisite for elective surgery) and quality assurance processes. The identified cases illustrate that operating room staff work as a team with shared responsibility and accountability for patient safety;^7^ therefore, surgeon performance data alone may not necessarily be useful in the case of minimising RSIs, particularly in cases of prolonged retention. We did find, though, that current team-based risk mitigation strategies, including counting, communicating, and documenting items used during surgery, are not always effective.^7^

### Need for Multidisciplinary Guidelines for Perioperative Practice

Like in many countries around the world, most facilities in Australia have incorporated the World Health Organization’s (WHO) Surgical Safety Checklist into routine practice in the operating room, with varying degrees of success.^53^ Although WHO has encouraged facilities to adapt the checklist to fit local practice, the checklist includes only one item specifically targeting prevention of RSIs, that is, during the “sign out” phase the “nurse verbally confirms with the team … that instrument, sponge and needle counts are correct (or not applicable)”. In Australia, the Australian College of Perioperative Nurses (ACORN) is the only professional body providing explicit guidance, in the form of standards for perioperative practice, related to the prevention of RSIs.^16^ We have not been able to identify any published equivalent guidance produced by the Royal Australasian College of Surgeons (RACS) or the Australian and New Zealand College of Anaesthetists (ANZCA) for their members. This may be because the responsibility for the management of accountable surgical items has historically been considered the domain of the perioperative nurse, despite the multidisciplinary team environment in which surgical procedures are typically conducted. It is therefore timely to consider the development of multidisciplinary guidelines for perioperative practice that are endorsed by the professional bodies of all disciplines that make up the team.

The cases analysed in our study highlight the importance of shared responsibility, particularly for communication and documentation, and for compliance with established processes to reduce the risk of harm. The cases also highlight varying outcomes in judicial determinations of alleged negligence in the advent of an RSI. However, as such, it appears that the “elaborate ritual”^33^ of manual counting and management of accountable items prescribed by ACORN in the national standards for the profession is not sufficient to prevent all incidents of RSIs from occurring. In all included case reports that explicitly discuss the count procedure, the procedures described correspond with current ACORN Standards for Perioperative Nursing in Australia.^16^ As such, the fact that these procedures were not sufficient to avoid the retention of surgical items is a relevant consideration for contemporary prevention and protective strategies. Our findings in this context align with the recent findings by Gunnar et al^54^ in their study of root cause analysis of RSI events, which found that a majority of incidents (64%) involved human factors issues (eg, staffing changes during shifts, staff fatigue), policy/procedure failures (eg, failure to perform methodical wound sweep) or communication errors.^54^

In addition, standard and usual processes outlined in ACORN Standards for locating missing items in the event of a discrepancy in the count, including immediately notifying the surgeon, requesting a thorough re-exploration of the wound, search of environmental surroundings and intra-operative imaging, do not provide a completely effective prevention strategy. This conclusion, derived from an analysis of case law, is supported not only by the literature but also by state government patient safety reports that point to procedural non-compliance as a key contributing factor to surgical item retention. This naturally leads us to consider the need to adopt newer, technologically advanced adjunctive strategies, particularly those with evidence of effectiveness.^20^,^21^,^54^ This strategy to improve detection and supplement counts, and the need for an evidence base in this area, was also highlighted by Hibbert et al.^50^ The continued persistence of RSIs across the world, including Australia, highlights the shortcomings of current prevention strategies in totally preventing this sentinel event and at the same time questions the assumption that an RSI is a never-event.

### Patient Engagement for Early Detection of Retained Surgical Items

The occurrence of never events, such as RSIs, undermines the trust and confidence that the public has in a healthcare system. Most facilities follow-up patients for signs and symptoms of infection. A survey of 462 internal medicine patients across five university hospitals in Finland^55^ found that when patients have positive health care service experiences, they participate more in ensuring their own safety during hospital care. This premise could naturally extend to post-hospitalisation patient safety practices. It is worth considering a longer postoperative follow-up period and investigation of all patient-reported outcome measures (PROMs), regardless of whether the symptoms reflect “usual” postoperative complaints (like surgical pain or surgical site infection) or are non-specific. Of course, the patient may not tell us at the time that something had been left behind. However, healthcare professionals need to improve the information and encouragement we give to patients, so patients can be more pro-active in their own postoperative safety practices,^55^ such as reporting signs and symptoms some of which could assist in identifying RSIs earlier in the post-discharge period.

Perhaps, RSI should become a routine differential diagnosis until ruled out when patients report postoperative complaints. This recommendation may serve as a useful outward indicator to patients that the healthcare system values their participation in improving the safety and quality of healthcare, is listening to their worries, and is concerned with their safety.

### Need for Globally Standardised Ontology and Taxonomy and Mandatory Reporting

The true incidence and prevalence of RSIs is difficult to accurately quantify due to the nature of reporting as well as the inconsistency in operational definitions and measurement indicators. For example, inconsistency in reporting near misses, that is, situations of an incorrect count where the RSI is subsequently located prior to wound closure or prior to the patient leaving the operating room. Furthermore, there are very little data on miscounts, that is, situations where the count is deemed correct at the end of the procedure, yet an RSI is identified later after the wound is closed and the patient has left the operating room, and in many cases, the hospital.

The original definition of RSIs in Australia was changed from “retained instruments or other material after surgery requiring re-operation or further surgical procedure” in 2002 to “unintended retention of a foreign object in a patient after surgery or other invasive procedure resulting in serious harm or death” in 2018.^56^ Serious harm is defined by the Australian Commission on Safety and Quality in Health Care as being permanent or long-term physical harm, permanent or long-term loss of function; shortened life expectancy, or the patient requiring life-saving surgical or medical intervention.^56^ This implies that if no serious harm or death results, then the incident does not need to be reported. This would also exclude near misses where the missing item was found before the wound was closed or the patient was transferred from the operating room. However, once again, this limits the opportunity to estimate true risk. 

By contrast, in the USA, the current Joint Commission definition is that “an unintended retained foreign object (URFO) [is] an object that is retained after skin closure has occurred following an invasive procedure”,^57^ that is, the definition is not limited to cases where the retention results in serious harm or death and does not specify that the patient has left the operating room. Contrary to this, the definition from the National Quality Forum, also in the United States, states, “ … the patient has been taken from the operating/procedure room” (pB-4). In the United Kingdom, the 2009 never event was called “retained surgical instrument postoperation”, then “retained instrument”, and finally in 2011, “retained foreign body postoperation”.^58^ The definitional inconsistency around the world has the potential to impact on the accuracy of indicators not only of actual RSIs but also of the true risk, making benchmarking problematic and contributing to the underestimation of the extent of the problem. Standardised data collection is important for accurately interpreting outcomes data.^59^ What is needed is a globally standardised ontology and taxonomy including operational definitions and clearly demarcated measurement indicators, and mandatory reporting based on these standard indicators.

### Open Access to Data

Once accurate data are captured, the data need to be stored and made accessible. Changes in healthcare and developments in information systems have seen an increase in the use of big data sets captured in large national databases, particularly in surgical research.^60^ Establishing a national or international registry for the tracking and surveillance of patients identified as having an RSI and those with a differential diagnosis of RSI would provide the opportunity for accurate estimates of the problem and of the risk; and may lead to global collaborative efforts to address this never event. Donabedian’s model of quality improvement posits that structure measures have an effect on process measures, which have an effect on outcome measures.^61^ Thus, registry data that include structure, process and outcome indicators would allow a more complete evaluation of current strategies for preventing RSIs as well as how we have moved forward to any sustainable improvements in reducing incidence and prevalence, which technically, should be zero.

### Limitations

We acknowledge the inherent limitations of using case law as a data source. First, in legal proceedings, the parties and their legal representatives argue their case and present the facts in a way that is likely to advance their claim and establish the necessary elements. In addition, when a judge is drafting their decision (judgment), the judge generally filters the detailed information presented at trial to only the facts that are material to the judicial reasoning process. This limits the case details that are publicly available for analysis. Second, the extent of information contained in the cases was a limitation. For example, some cases contained very detailed factual information, including antecedents and human costs of living with an RSI, whereas others simply provided a brief overview of the outcomes limited to less than a page of information. Varying degrees of information were provided about counts and contemporaneous record keeping.

The study was limited to cases that were available by searching publicly accessible databases, which resulted in our systematic review identifying only a small number of cases. In addition, most cases reviewed were procedural; as such, some of the factual circumstances, which would have been recorded in a report of the full trial decision were missing.

Further to this, more than 95% of Australian medical litigation is settled (resolved) through negotiation or discontinued before a final judicial determination,^7^ and the outcomes of the fact and details of the settlements are usually confidential. This limits the ability to engage in additional fine-grain analysis that would have been undertaken in this review had these cases gone to trial.

Despite this, our critical analysis of these cases expands upon many of the issues raised in the government reports in terms of antecedents and human costs of living with RSI.

## Conclusion

An RSI can be discovered days, weeks, months, or years after the original operation, usually following the development of patient symptoms. Unintentional retention of surgical items has been recognised as such for more than a decade by the Australian Commission on Safety and Quality in Health Care as an event that causes serious harm to patients and threatens society’s perception of the Australian healthcare system. Mandatory federal reporting of RSIs as a sentinel event allows researchers to track the frequency of these events, while state reporting provides some anecdotal evidence as to specific case studies. Despite this, there is a current dearth of online, publicly available information that provides clear insights into the nature and extent of RSIs.

Our case law analysis supplemented data from state government reports that examine the immediate physical complications impacting the patient. Our analysis highlighted patient circumstances related to the aftermath of not only living with an RSI but also psychosocial and emotional distress once a patient becomes aware of living with an RSI, information that only comes to light following the delayed discovery of the retained item.

The case law related to RSIs to date is very limited, with only nine civil cases and one coronial case dealing with this issue since the early 1980s, which is explained by the small number of claims that proceed to publicly available judicial determination. Further research could extend to reviewing trial transcripts, as well as de-identified insurance claims and settlement documents (if not subject to a confidentiality agreement). Nevertheless, our review of the decided cases indicates that current forms of risk management to minimise or eliminate the incidence of this sentinel event, including standards-based professional perioperative practice and mandatory reporting of adverse events, are not always effective in preventing retention. Additional measures, including newer technologies for detection, should be explored, and those with clear evidence of effectiveness should be deployed where resources permit. In addition, estimates of the true risk of RSI in Australia can be improved by more standardised and consistent reporting of risk of RSIs; not just reporting of actual events but also near misses; and consistency across jurisdictions about the definition of RSIs, including whether it is limited to cases involving serious harm or death, and the timing of when an item is considered retained (for example, before or after wound closure; before or after leaving the operating room). Finally, this study has presented a starting point for a call to action for a consistent methodology, ontology and taxonomy for mining data from case law to inform better understanding of RSIs that can contribute to better estimates of the global nature and extent of the risk, as well as the problem.

## Data Sharing Statement

The data (case reports) underpinning the results presented in this study are available from publicly available databases and repositories. Details of these databases and repositories are included in the Supplementary Materials related to the search strategy.
